# Artificial Enamel

**Published:** 1907-10-15

**Authors:** R. B. Tuller


### ARTIFICIAL ENAMEL.

BY R. B. TULLER, D.D.S.

As time goes on there is something more to be noted and said about artificial enamel, that substance classed as a silicate cement which has so recently been introduced as filling material for the teeth

Several makes of silicate cements have been before the dentists of this country, all claiming excellent virtues or qualities in one way and another, and especially the essential one of being durable as well as translucent and life-like. Some of these claims have not been borne out when put to practical tests, the result being that a prejudice has been aroused with many against the whole class, when there certainly are rare virtues in some of them. The one that the writer has had most experience with, and the only one that he has had success with, as yet, is called artificial enamel (Ascher's) and the name is very aptly applied.

This particular product has been before the dental profession of Europe, or some parts of it, for about five years; but, except in the hands of a very few, perhaps, it has not been before the profession here and subject to practical tests and usage much more than two years.

Two years in the hands of a large number of operators has shown something encouraging, if it has not been all that was hoped for, or could be wished for. It has proved that the new material is a valuable adjunct in the way of cement, if it may not yet be accepted as an ideal substance with reasonable claims of durability as a filling for a large variety of cavities.

The testimony of a large number of users would indicate that it can be relied upon for perhaps more than average durability; and, with a more intimate knowledge of its peculiarities and the essential steps in compounding the two ingredients, liquid and powder, and also of manipulative precautions, the operator may find much closer approximation to the looked-for ideal.

If a filling material in practical use in the mouth or in many mouths stands up reliably and without apparent waste or disintegration for two years, it is quite reasonable to expect it to do service for another two years. It should show clear evidence if any disintegration is under way; finding none after such time, the "table of expectation" goes up several points.

Remembering the varied results of mixing oxy-phosphate cements from the same "batch," and using simialr proportions as far as could be observed, and remembering that artificial enamel is a cement, it would not be strange if some, out of a number of fillings, should prove defective, and soon show signs of breaking down. But that need not imply anything against the material *per se*, but simply that the mixing or manipulation had not been properly done in one or several particulars, possibly. One may so manipulate gold or amalgam that fillings are faulty and fail.

In making porcelain inlays most operators are unfortunate enough to have some of them come out, through some fault in the mix of cement which fails to hold. Not infrequently another mix from the same material and seemingly under exactly like condition, will hold the reset inlay indefinitely. Occasional failures to make them stay does not discountenance inlay work; and neither should some failures in artificial enamel discourage an operator as to further effort along that line, with better understanding from experience.

Owing to lack of knowledge of exact proportions, or the impracticability of getting those exact proportions, if we knew them, and the several conditions that have to be taken into account in mixing cements of any kind, such as time in mixing and time in handling after the mix (and before crystallization demands no further interference), temperature and perhaps humidity of the atmosphere, we can attain *perfect* results only by accident, but may approximate closely by experience and observation. Only by this means, coupled with the data provided by those who have gone before, can near perfection be attained.

In the first experiments with Ascher's enamel one would do well first to read the latest instructions—the later ones differing considerably from the first that accompanied the material—the result of greater experience—and then fill cavities in teeth out of the mouth, although the conditions of temperature are different, which should perhaps be taken into account. The producer of the enamel calls attention to t[illegible] is and says experiments out of the mouth should be brou[illegible]ht up to the normal temperature of the body—or in round figures to 100 degrees F.—and be kept dry until crystallization has been effected.

But the unexperienced operator can learn many of the peculiarities of the enamel as to mixing and filling cavities out of the mouth without concerning himself about the temperature, except to remember that any cement will crystallize quicker, if that is desired, under warm instruments.

It is perhaps more difficult to give all the intimate and peculiar directions, how to handle the enamel in question in a variety of cavities, than almost any other material; and while it is a cement, it is different in many ways from any other, or from oxy-phosphate cements. In one particular, Ascher's enamel may be manipulated, within limits, up to the very time it becomes too hard to do so. In fact, it is essential to do the final finishing and burning with oiled burnishers just before it becomes too hard to yield.

If possible, a filling should be molded to the form desired, after first forcing the material to sure contact with all the cavity walls, under cuts and margins, and then skillfully trimmed to remove all surplus overlapping beyond margins. This may be done when material is yet soft by the instrument used in putting it to place, or another if more convenient; but as the contouring progresses and the filling grewing harder there will be more trimming to be done that can only be done satisfactorily with a thin, long-pointed sharp blade. (oiled) like a slender lancet blade. If the filling is kept level with margins in this way and the burnishing with the oiled burnishers as per instructions (which, by the way, must be done without much delay when the material is hardening rapidly), no disking or stripping will be necessary, and this is the most desirable way. The use of grit disks and strips should be avoided if possible for the reason that their use leaves a scratched and undesirable surface; hard to bring later to a high polish, which is essential if the best appearance in translucency and in shade are to be attained. High polish also stands for more certain durability. As to shades, one may have selected the best to correspond with the particular tooth involved and then spoil the effect by disking or stripping with a gritty disk or strip, unless a high smooth polish can be secured after. This is not easy; for when this enamel is once hard it is very hard, and in the nature of it does not then take a fine polish without a long, tedious effort, depending somewhat upon how hard it has become when the polishing is undertaken. There is a time before it is absolutely hard (but it don't tarry very long) when it may be cut down with disks and strips (always oiled with vaseline) and then polished with the highly polished celluloid or terra plastica strips and disks. But at that particular period of hardness, or lack of it, it is an easy matter to allow disks and strips to cut in too deep and destroy the contour one has taken pains to perfect; and at the same time it is too hard to do any further modeling to bring back to shape. It is altogether desirable, as before said, to do all finishing *without* the use of the usual grit disks and strips, no matter how fine. When the material is once too hard the celluloid disks and strips are of little use in getting a high polished surface.

In a proximal filling the celluloid high polished strips serve as a matrix and leave a surface equal to their own on the filling; but unless this matrix is held tight to the margins of the cavity, it will be easily understood that the material will squeeze out between the margins of cavity and leave an overlap, which should be removed; and sometimes this overlap blends so perfectly with the tooth enamel that it is difficult to detect. If left, it would in due time break away and leave the filling standing out beyond the cavity margin. When this has occurred, stripping with sharp strips may be necessary—or disking—at another time, of course, doing the final finish as best one can with the finest —always oiled.

It is often found that any burnisher—not metallic—is too large and clumsy to reach certain parts, and steel may be used by interposing the end of one of those thin flexible celluloid strips, thus keeping the metal from contact with the filling, but permitting of burnishing and modeling with a high polished surface against the filling—again, always oiled enough to prevent the material from sticking.

When these strips are used as a matrix they should be just slightly oiled to prevent material from sticking.

Instead of separating teeth where, for instance, there is to be considerable contour between two bicuspids, or a bicuspid and molar, to allow for matrix, and coming back to contact after matrix is removed, the writer does as he often has done with amalgam when conditions will permit, and that is, pack against a matrix and then before material is hard slide out the matrix and with force occlusally applied, and the use of burnishers to assist in modelling, if need be, bring about a proper contact with the neighboring tooth, or even between two proximating fillings put in at the same time.

Separating teeth is a very unpleasant experience for the patient, if not extremely painful; and if the work can be accomplished properly without, it certainly should be done, and it can be in many instances.

With the enamel it is easy, comparatively, yet requiring some skill and judgment. Slightly oil and place in the matrix. It may be held to place by any one of several devices for the purpose, perhaps. Or it may be just slipped in and left until the material is packed in, the latter being modeled by bending the strip around on either side with a burnisher. If the enamel is hardened some the matrix may be pushed out of the way and the overlapping surplus removed, the matrix is again pressed around the filling and the surplus, if any, removed again. Or the matrix may be slipped out entirely and modeling and trimming done, and then matrix may be replaced, and pressed again around the filling. Now, before filling becomes too hard attend to the occlusal contouring, removing the overlap into the occlusal irregularities with sharp spoon and other excavators, and finally, before filling is too hard, remove the matrix and exert a pressure occlusally with a suitable ball or ovel burnisher, to cause the filling to bulge out to fill the space that was occupied by the matrix strip. If this is done at the right time the contact will be correctly made and contour be entirely symmetrical, or easily regulated with the proper burnisher.

Speaking of these contour fillings of enamel in both bicuspids and molars, the writer has quite a number of large ones under observation and some have been fully two years in place without signs of giving way from any cause. In some instances, where a failure has occurred, the fault has undoubtedly been in mixing or subsequent manipulation. Failure from such causes is not long in becoming manifest, and, with the writer, the second filling has proven satisfactory. While they remain in the tooth they do not leak, but stick to the walls and preserve. If there is any shrinkage at all of this material it shrinks slightly upon itself and never cleaves from the wall of the tooth, leaving a ditch, as shrinking amalgam does; at least, so far as the observation of the writer goes.

A word about shades. The shade guide provided with the material is far from being satisfactory. A shade guide should be of some such a nature as the guides for porcelain, easy to place in contact with the tooth for close comparison. And further, some little idea might be given in instructions as to combining shades, though it must of course depend even then largely on the artistic eye or sense of the operator as does the selection with the guide. One thing may be remarked with some certainty: that most operators will make the mistake oftener of selecting a shade too light for the case than too dark. Numbers two and four and a combination of these two shades are perhaps more often demanded than any others, or a pearl gray and a dark yellow.

The fine artistic ability or sense of an operator may be brought out by mixing two colors (separately), as a suitable yellow for the neck and a pearl gray for the incisal third or half, and gradual blending of the two between. The blend may be made very satisfactorily in the following manner: While the operator mixes one color his assistant should mix the other. When ready the yellow, or cervical, should be placed first in the proper proportion; then the other placed quickly, a little dragging of the two shades back and forth a little at the joining of the two, only while material is soft the blend may be made very natural.

Matching up the shades of an incisor, for instance, or any exposed tooth, may be done to the greatest perfection. The writer has done it in case of incisors so that no one could detect repair, not even dentists asked to make the examination. In other cases, not so perfect as that just stated, the match as yet has been so close that it would escape the observation of any one without a very close examination intent on discovery. These fine matchings and shadings may be undone if, as has before been stated, finish is attempted by the use of even very fine cuttle fish disks; therefore, the operator should aim to so move and manipulate and trim with a sharp oiled blade, cutting always toward the cavity margins, followed by smoothing with suitable burnishers. Use steel burnisher with the celluloid strip held in left hand interposed between instrument and filling.

Oil (vaseline) should be used at the right time and place, but not in any way to become incorporated in the filling material before ready to surface. Oil no instrument used in packing material into cavity; nor until ready to smooth up the surface after modeling and shaping.—*American Journal.*

